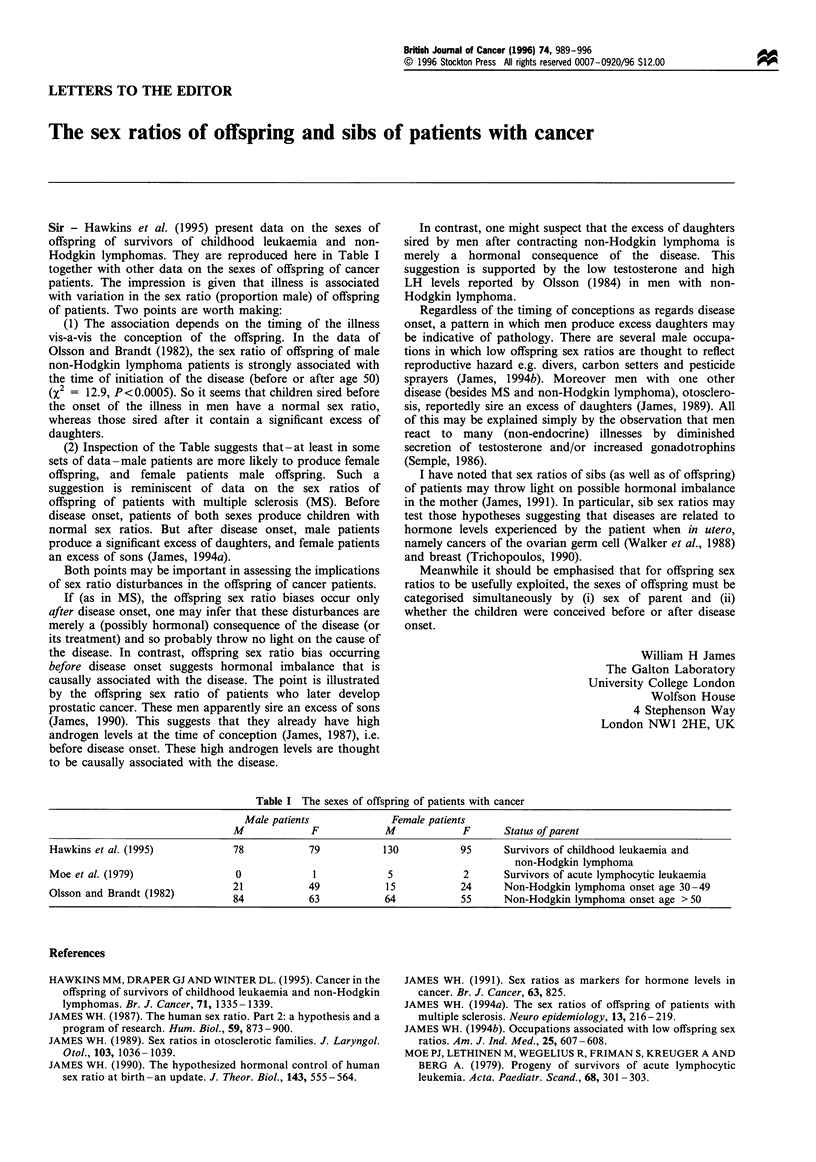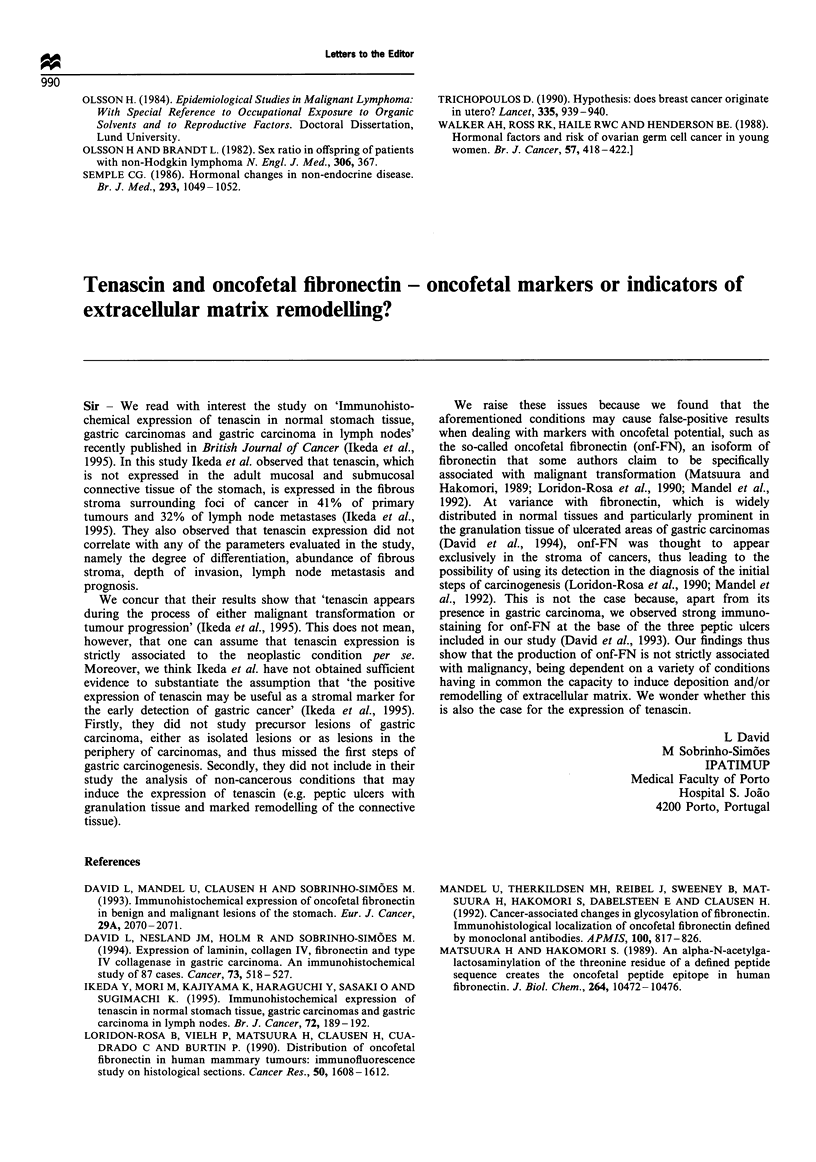# The sex ratios of offspring and sibs of patients with cancer.

**DOI:** 10.1038/bjc.1996.471

**Published:** 1996-09

**Authors:** W. H. James


British Journal of Cancer (1996) 74, 989-996

© 1996 Stockton Press All rights reserved 0007-0920/96 $12.00

### LETTERS TO THE EDITOR

### The sex ratios of offspring and sibs of patients with cancer

Sir – Hawkins *et al.* (1995) present data on the sexes of offspring of survivors of childhood leukaemia and non-Hodgkin lymphomas. They are reproduced here in Table I together with other data on the sexes of offspring of cancer patients. The impression is given that illness is associated with variation in the sex ratio (proportion male) of offspring of patients. Two points are worth making:

(1) The association depends on the timing of the illness vis-a-vis the conception of the offspring. In the data of Olsson and Brandt (1982), the sex ratio of offspring of male non-Hodgkin lymphoma patients is strongly associated with the time of initiation of the disease (before or after age 50) ($\chi^2 = 12.9$, $P<0.0005$). So it seems that children sired before the onset of the illness in men have a normal sex ratio, whereas those sired after it contain a significant excess of daughters.

(2) Inspection of the Table suggests that – at least in some sets of data – male patients are more likely to produce female offspring, and female patients male offspring. Such a suggestion is reminiscent of data on the sex ratios of offspring of patients with multiple sclerosis (MS). Before disease onset, patients of both sexes produce children with normal sex ratios. But after disease onset, male patients produce a significant excess of daughters, and female patients an excess of sons (James, 1994*a*).

Both points may be important in assessing the implications of sex ratio disturbances in the offspring of cancer patients.

If (as in MS), the offspring sex ratio biases occur only *after* disease onset, one may infer that these disturbances are merely a (possibly hormonal) consequence of the disease (or its treatment) and so probably throw no light on the cause of the disease. In contrast, offspring sex ratio bias occurring *before* disease onset suggests hormonal imbalance that is causally associated with the disease. The point is illustrated by the offspring sex ratio of patients who later develop prostatic cancer. These men apparently sire an excess of sons (James, 1990). This suggests that they already have high androgen levels at the time of conception (James, 1987), i.e. before disease onset. These high androgen levels are thought to be causally associated with the disease.

In contrast, one might suspect that the excess of daughters sired by men after contracting non-Hodgkin lymphoma is merely a hormonal consequence of the disease. This suggestion is supported by the low testosterone and high LH levels reported by Olsson (1984) in men with non-Hodgkin lymphoma.

Regardless of the timing of conceptions as regards disease onset, a pattern in which men produce excess daughters may be indicative of pathology. There are several male occupations in which low offspring sex ratios are thought to reflect reproductive hazard e.g. divers, carbon setters and pesticide sprayers (James, 1994*b*). Moreover men with one other disease (besides MS and non-Hodgkin lymphoma), otosclerosis, reportedly sire an excess of daughters (James, 1989). All of this may be explained simply by the observation that men react to many (non-endocrine) illnesses by diminished secretion of testosterone and/or increased gonadotrophins (Semple, 1986).

I have noted that sex ratios of sibs (as well as of offspring) of patients may throw light on possible hormonal imbalance in the mother (James, 1991). In particular, sib sex ratios may test those hypotheses suggesting that diseases are related to hormone levels experienced by the patient when *in utero*, namely cancers of the ovarian germ cell (Walker *et al.*, 1988) and breast (Trichopoulos, 1990).

Meanwhile it should be emphasised that for offspring sex ratios to be usefully exploited, the sexes of offspring must be categorised simultaneously by (i) sex of parent and (ii) whether the children were conceived before or after disease onset.

William H James
The Galton Laboratory
University College London
Wolfson House
4 Stephenson Way
London NW1 2HE, UK

**Table I** The sexes of offspring of patients with cancer

| | *Male patients* | | *Female patients* | | |
|---|---|---|---|---|---|
| | *M* | *F* | *M* | *F* | *Status of parent* |
| Hawkins *et al.* (1995) | 78 | 79 | 130 | 95 | Survivors of childhood leukaemia and non-Hodgkin lymphoma |
| Moe *et al.* (1979) | 0 | 1 | 5 | 2 | Survivors of acute lymphocytic leukaemia |
| Olsson and Brandt (1982) | 21 | 49 | 15 | 24 | Non-Hodgkin lymphoma onset age 30–49 |
| | 84 | 63 | 64 | 55 | Non-Hodgkin lymphoma onset age >50 |

### References

HAWKINS MM, DRAPER GJ AND WINTER DL. (1995). Cancer in the offspring of survivors of childhood leukaemia and non-Hodgkin lymphomas. *Br. J. Cancer*, **71**, 1335–1339.

JAMES WH. (1987). The human sex ratio. Part 2: a hypothesis and a program of research. *Hum. Biol.*, **59**, 873–900.

JAMES WH. (1989). Sex ratios in otosclerotic families. *J. Laryngol. Otol.*, **103**, 1036–1039.

JAMES WH. (1990). The hypothesized hormonal control of human sex ratio at birth – an update. *J. Theor. Biol.*, **143**, 555–564.

JAMES WH. (1991). Sex ratios as markers for hormone levels in cancer. *Br. J. Cancer*, **63**, 825.

JAMES WH. (1994*a*). The sex ratios of offspring of patients with multiple sclerosis. *Neuro epidemiology*, **13**, 216–219.

JAMES WH. (1994*b*). Occupations associated with low offspring sex ratios. *Am. J. Ind. Med.*, **25**, 607–608.

MOE PJ, LETHINEN M, WEGELIUS R, FRIMAN S, KREUGER A AND BERG A. (1979). Progeny of survivors of acute lymphocytic leukemia. *Acta. Paediatr. Scand.*, **68**, 301–303.

OLSSON H. (1984). *Epidemiological Studies in Malignant Lymphoma: With Special Reference to Occupational Exposure to Organic Solvents and to Reproductive Factors.* Doctoral Dissertation, Lund University.
OLSSON H AND BRANDT L. (1982). Sex ratio in offspring of patients with non-Hodgkin lymphoma *N. Engl. J. Med.*, **306**, 367.
SEMPLE CG. (1986). Hormonal changes in non-endocrine disease. *Br. J. Med.*, **293**, 1049-1052.
TRICHOPOULOS D. (1990). Hypothesis: does breast cancer originate in utero? *Lancet*, **335**, 939-940.
WALKER AH, ROSS RK, HAILE RWC AND HENDERSON BE. (1988). Hormonal factors and risk of ovarian germ cell cancer in young women. *Br. J. Cancer*, **57**, 418-422.]